# Bariatric and metabolic surgery medical tourism: the compelling need for regulation through transnational collaboration

**DOI:** 10.1136/bmjgh-2025-019546

**Published:** 2025-07-15

**Authors:** Jessica McGirr, Edward W Gregg, Deborah A McNamara, Grace O’Malley

**Affiliations:** 1 Obesity Research and Care Group, School of Physiotherapy, Royal College of Surgeons in Ireland, Dublin, Ireland; 2 School of Public Health, Imperial College London, London, UK; 3 School of Population Health, Royal College of Surgeons in Ireland, Dublin, Ireland; 4 Department of Colorectal Surgery, Beaumont Hospital, Dublin, Ireland; 5 Royal College of Surgeons in Ireland, Dublin, Ireland; 6 Children's Health Ireland at Temple Street, Dublin, Ireland

**Keywords:** Surgery, Nutritional and metabolic disorders, Global Health, Health policy, Public Health

**SUMMARY BOX**

- The rising prevalence of obesity coupled with globalisation of healthcare has resulted in an increasing number of people travelling to access bariatric and metabolic surgery.
- Bariatric and metabolic surgery medical tourism remains largely unregulated creating risks for patients and for healthcare systems within home and destination countries.
- Regulating the bariatric medical tourism industry through transnational multi-actor collaboration is essential to protect health and health equity.

## Introduction

 Obesity is a chronic disease defined by the WHO.^1^ The management of obesity involves a multifaceted approach including lifestyle, pharmacological, endoscopic and/or surgical management. The high prevalence of obesity coupled with healthcare resource constraints and increased globalisation has resulted in more people accessing obesity treatment outside their home country. To meet the higher demand for obesity management medicines (OMM), a concerning market providing unregulated OMM access has emerged, which poses unique risks and regulatory and ethical challenges.^2 3^ Some data suggests that increased use of pharmacotherapy may result in a reduction in bariatric and metabolic surgery (BMS), though the number of people travelling abroad to access BMS continues to rise.^4 5^ The term bariatric and metabolic surgery medical tourism (BMT) is used to describe this phenomenon.^6^ In the absence of reliable data, the extent of this industry is difficult to ascertain. The wider medical tourism industry is worth more than $400 billion annually with anticipated annual growth of 25%.^7^ Despite the estimated size of the BMT industry, it remains largely unregulated. This commentary focuses on the BMT industry through a global health lens, outlining the opportunities and risks it poses for individuals and for health systems. The need for regulation is highlighted, and recommendations to support regulatory processes are proposed. Key terminology is defined in table 1.

**Table 1:** Intended meaning of key terms and abbreviations

Term/ abbreviation	Intended meaning
Obesity	A chronic disease characterised by ‘abnormal or excessive fat accumulation that presents a risk to health’.^1^.
Bariatric and metabolic surgery (BMS)	Surgical intervention for the management of obesity and treatment of obesity-related complications.
Bariatric and metabolic surgery medical tourism (BMT)	Travelling outside of one’s local healthcare services to undergo bariatric and metabolic surgery.
Patient	Person living with obesity undergoing bariatric and metabolic surgery.
Provider	The surgical and wider healthcare team involved in the delivery of bariatric and metabolic surgery, including perioperative care.
Home country	The country in which people reside and travel from to undergo bariatric and metabolic surgery.
Destination country	The country to which people travel to undergo bariatric and metabolic surgery.
EASO	The European Association for the Study of Obesity.
IFSO	International Federation for the Surgery of Obesity and Metabolic Disorders.
ECPO	The European Coalition for People living with Obesity.

### The patient: opportunity

The BMT industry presents an opportunity for people living with obesity. The benefits of BMS in the treatment of obesity are well established, with high rates of remission of obesity-related complications observed postoperatively.^8^ Inadequate provision of BMS within public health systems and long waiting lists are commonly cited reasons for seeking surgery outside domestic healthcare systems.^7 9^ In addition to quicker access, cheaper costs are another driver for BMT.^7 9^ The out-of-pocket cost for private BMS in the UK is approximately £10 000–£15 000, while surgery can be accessed in countries such as Turkey at a cost of £2500–£4500.^10^ Quicker and cheaper access to surgery may benefit patients; however, it is not without risk.

### The patient: risks

The safety, ethical and legal risks for patients using an unregulated BMT industry are significant. The largest global survey to date of BMT providers highlighted that most patients (71%) self-refer for surgery.^7^ This creates potential risk related to inappropriate self-referral of individuals without appropriate medical indication to undergo major surgery. Indeed, ineligibility for BMS in one’s home country is cited by patients as a reason for accessing BMT.^9^ The absence of regulation of BMT marketing with no guaranteed information transparency creates additional risk. A descriptive analysis of online websites by BMT providers highlighted that just one of 34 centres provided information on complication rates.^11^ Another risk is the lack of preoperative and long-term nutritional, psychological and other medical follow-up. Multidisciplinary care is an integral part of appropriate case selection and is often lacking.

When considering adverse outcomes, including anastomotic leakage, sepsis and even death, equally concerning is the absence of regulation to ensure that only accredited procedures are performed by appropriately qualified providers. Further concern arises in the context of medical tourism ‘packages’ in which patients are offered multiple procedures within the same trip. There are often financial incentives used in the marketing of surgical ‘packages’; however, such practice is not evidence-based and carries increased risk of complications.

These considerations raise ethical and safety concerns, whereby the legal position of patients seeking redress should be considered. This is another area within the industry that lacks regulation and clarity. The pressing need to address this is supported by the results of a global survey of BMT providers in which 30% believed the consent process was ‘inappropriate’ and 14% believed that patients were personally responsible for surgical complications.^7^ The need to regulate the BMT industry to mitigate these safety, ethical and legal risks for patients is essential.

### The home country: risks and opportunities

In addition to patient considerations, the impact of the BMT industry on healthcare resources and the economy of the home country must be considered. BMS is recognised as a cost-effective intervention for obesity.^5^ Health economic analyses have demonstrated the cost-effectiveness of BMS in the treatment of type 2 diabetes for example.^8^ Single-payer healthcare systems may benefit from patients accessing surgery through the BMT industry both financially and through reduction in waiting lists.^8^ This potential for positive economic impact is not without risk. An area of great concern for home countries is the cost and resources incurred in providing postoperative care and managing complications for those returning from BMT.^12^ Several countries have reported significant impact of such presentations on local healthcare systems.^13 14^ Regulation is warranted to maximise benefit and minimise harm to the limited healthcare resources of home countries.

### The destination country: risks and opportunities

Managing the risks and opportunities created by BMT for destination countries is equally pressing. The flow of medical tourists is dominated by travel from high income to low- and middle-income countries (LMICs).^12 15^ Data on patient flow in the BMT industry largely depends on descriptive analysis, with recent reports of Poland, Turkey and Mexico having the greatest number of clinics offering BMS globally.^11^ It is imperative that regulation within destination countries is implemented to ensure the healthcare access needs of local populations are not compromised by the economic allure of providing BMT. A medical tourism scoping review published by the Organisation for Economic Cooperation and Development describes high government motivation to invest in healthcare workers and infrastructure to boost the medical tourism trade.^12^ This creates a risk for local populations where investment is potentially diverted from basic public health services and healthcare infrastructure towards the private sector.^12^ The resulting financial incentive could potentially drive local surgeons to favour working in BMT, diverting staff away from essential services such as trauma and emergency surgery.

However, there is potential for the BMT industry to positively impact local populations. A proportion of revenue generated as a direct result of BMT could be reinvested back into the public healthcare system, as was implemented in Cuba.^12^ Without transnational regulation of the BMT industry however, the current system risks worsening healthcare access and inequity within LMICs.

### Current governance of bariatric and metabolic surgery medical tourism

Despite the estimated size and identified risks of the BMT industry, it remains largely unregulated. In a global survey of nearly 400 BMT providers, over half of respondents had never heard of international healthcare accreditation, with such accreditation in place in less than 20% of hospitals.^7^ Governance and policy that exists at a national level is influenced by provider and governmental priorities.^15^ Mexico recently published a national consensus statement to promote safe practice within the BMT industry.^16^ It is noteworthy, however, that most (93%) contributors to this consensus statement were surgeons working exclusively in the private sector.^16^ Reliance on private practitioners for national guidance is potentially concerning. The comorbidity and socio-economic profile of patients availing of private healthcare is not representative of the entire population and underestimates surgical risks. There is concern that morbidity and mortality are underestimated by the private sector as patients generally present to larger, public hospitals when complications arise after early discharge. Additionally, highly relevant stakeholders including patients and members of the multidisciplinary team such as dieticians were excluded from the development of this consensus statement and best practice in consensus guideline development was not implemented.^16 17^ The current regulation of practice is inadequate and highlights the need for transnational collaboration among all sectors to implement regulation.

### Resistance to the regulation of the bariatric and metabolic surgery medical tourism industry

Considering these conflicting priorities, resistance to regulation of the industry is anticipated. Providers of BMT and national governments may resist the implementation and/or enforcement of regulation that would itself be costly or that might reduce profitability.^15^ Governmental bodies will be acutely aware of BMT provider exit capacity, which may limit their willingness to enforce regulation. At the provider level, 2.4% of respondents to the global BMT survey expressed a belief that the industry should be banned and may therefore be hesitant to support a regulatory process.^7^ Despite anticipated resistance against regulation, it must be recognised that the BMT industry will continue to grow and, while providing access to treatment, create risks that must be mitigated.

### Collaboration and transnational regulation

The BMT industry requires regulation to provide safe, legal and ethical practice while protecting the health of local populations. In Europe, leading international associations (EASO, IFSO and ECPO) have published recommendations for safe practice in BMT.^6^ While national and regional governance is essential, the complex landscape of BMT and the anticipated challenges for individuals engaging with regulatory structures in destination countries call for the establishment of a global forum of stakeholders to develop transnational regulation. This complex and iterative regulatory process must involve collaboration between trade, health, advocacy and legal representatives. Identified actors and proposed regulation priorities are outlined in table 2. International surgical bodies such as IFSO or existing assurance bodies, such as the Joint Commission International, could support quality assurance of BMT surgical units through a provider-funded accreditation process. Mandatory provision of understandable patient- and population-level information could form part of this accreditation to support patients in making more informed and safer decisions.

**Table 2:** Identified actors and key priorities for transnational collaborative regulation

Identified actors	Key regulatory priorities
World Trade Organisation (WTO) WHO European Union (EU) Organisation for Economic Co-operation and Development (OECD) National governmental representation; Department of Health Department of Foreign Affairs International experts providing BMS (public and private sector representation is essential); International Federation for the Surgery of Obesity and Metabolic Disorders (IFSO) American Society for Metabolic and Bariatric Surgery (ASBMS) The European Association for the Study of Obesity (EASO) World Obesity Patient representation European Coalition for People Living with Obesity (ECPO)	Restriction on marketing within the industry and ensuring patient access to transparent understandable information. Unbiased information sources that are independent of the providers/direct beneficiaries of the industry should be made available. Standardisation of perioperative management aligned with international clinical practice guidelines. Emphasis should be placed on preoperative assessment, multidisciplinary team management and postoperative follow-up. Sharing of information across health systems with the provision of standardised discharge documentation incorporating a standardised data set is essential.^18^ Standard setting for BMS with development of accredited international surgical training programmes. Implementation of a transparent accreditation process to guarantee surgical procedures are undertaken by appropriately trained providers in safe settings. Listings of appropriately regulated and certified BMS centres should be transparent and accessible. Development of policy that protects patients and healthcare systems in the case of legal redress. 30 day outcome data to accurately capture morbidity and mortality data.

## Conclusion

Increased obesity prevalence, healthcare globalisation and health system resource constraints are resulting in growth of the BMT industry. This unregulated industry presents opportunity for quicker access to effective treatment for individuals with obesity but carries potential safety, ethical and legal risks. The economy and healthcare resources of both home and destination countries may benefit financially from BMT, but the potential for unintended negative consequences and widening health inequity are significant. Establishing regulation through transnational collaboration is essential to protect health and health equity.

## Data Availability

There are no data in this work.
